# Can COVID‐19 cause severe neutropenia?

**DOI:** 10.1002/ccr3.3369

**Published:** 2020-10-26

**Authors:** Patricia López‐Pereira, Isabel Iturrate, Rafael de La Cámara, Laura Cardeñoso, Adrián Alegre, Beatriz Aguado

**Affiliations:** ^1^ Hematology Department Hospital Universitario La Princesa Madrid Spain; ^2^ Microbiology Department Hospital Universitario La Princesa Madrid Spain

**Keywords:** coronavirus, COVID‐19, neutropenia, G‐CSF

## Abstract

This is the first case of acquired severe neutropenia in the context of COVID‐19 reported to date. This could illustrate another less frequent hematological disorder related to this novel viral infection.

## Graphical abstract

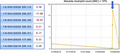

## 1 INTRODUCTION

The severe acute respiratory syndrome coronavirus 2 (SARS‐CoV‐2) produces a broad spectrum of respiratory symptoms, from mild to pneumonia and acute respiratory distress syndrome. Coronavirus disease (COVID‐19) is associated with an inflammatory syndrome that may increase damage to the lungs. Several hematopoietic and hemostatic abnormalities have been described, as well as autoimmune disorders.^1^, ^2^

## 2 CASE HISTORY

We report a 33‐year‐old woman, a surgical nurse, with no relevant medical history (although her sister was diagnosed with Hodgkin lymphoma), who was admitted to the Hematology Department at our hospital with severe neutropenia, asthenia, and enlarged lymph nodes 11 days after being diagnosed with COVID‐19.

On April 1, the patient presented at her family doctor (FD) with a sore throat, asthenia, and myalgia. SARS‐CoV‐2 polymerase chain reaction revealed positivity on oropharyngeal swabs obtained by the FD on April 2. She did not receive antiviral treatment, only acetaminophen when needed, and was discharged to her home to remain in isolation. Initially, her symptoms improved, but on April 13, she presented to the emergency department because of asthenia, gum swelling, and chills without fever or respiratory symptoms, such as shortness of breath or cough.

Her vital signs, including oxygen saturation, were normal, and physical examination showed mild enlargement of the cervical, axillary, and inguinal lymph nodes without other pathological findings.

**FIGURE 1:**
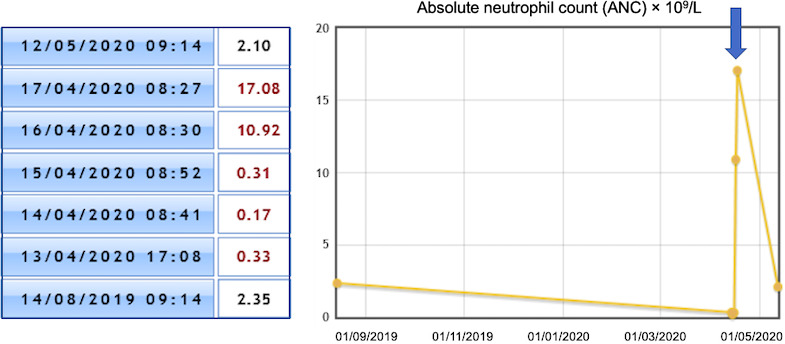
Absolute neutrophil count evolution. The blue arrow points a single dose of G‐CSF administration on 14/04/2020

### 2.1 Investigations and treatment

Blood counts showed mild leukopenia (3.07 × 10^9^/L) with severe neutropenia (0.33 × 10^9^/L) and eosinopenia (0.05 × 10^9^/L) with a normal lymphocyte count, hemoglobin level, and platelet count. No morphological abnormalities were observed in the peripheral blood smear. Blood biochemistry assessment showed an increased level of C‐reactive protein (2.51 mg/dL; normal, <0.5 mg/dL). Her lactate dehydrogenase level, liver function, and renal function were normal. Her interleukin‐6 level was 20 pg/mL (normal, <7 pg/mL). She had a fibrinogen level of 603 mg/dL with a normal D‐dimer level. Her chest radiography was normal, and the pregnancy test result was negative.

A new oropharyngeal swab for the SARS‐CoV‐2 test was performed on April 14, which continued to show positivity. The SARS‐CoV‐2 qualitative serology [immunoglobulin(Ig)G + IgM] was positive. Serology for human immunodeficiency virus (HIV), hepatitis C virus, toxoplasma, surface antigen of the hepatitis B virus, and hepatitis B core antibody tested negative. The titer of hepatitis B surface antibody was 24.87 mUI/mL. The cytomegalovirus, Epstein‐Barr virus (EBV), and parvovirus B19 serologies showed positivity for IgG.

Following these results, the patient was admitted to the Hematology Department, without any specific treatment for COVID‐19, to undergo complementary studies.

On April 14 (day 1 of admission, day 13 of infection), blood tests showed an absolute neutrophil count (ANC) of 0.17 × 10^9^/L. Her levels of iron, folate, and vitamin B12 were normal. Autoimmune screening (antinuclear antibodies, antineutrophil antibodies, antithyroid antibodies, and direct antiglobulin test) revealed negativity, and serum protein electrophoresis and Igs were normal.

Bone marrow aspirate showed normal cellularity in number and proportions, with no dysplastic changes or increase in the number of blast cells. The myeloid:erythroid ratio was 2.8. The granulocyte line showed normal maturation. There was no evidence of hemophagocytosis. Flow cytometry and cytogenetic analysis showed no pathological findings, with a normal karyotype.

Whole‐body computed tomography did not show any significant lymph nodes or visceral enlargement. A single dose of granulocyte colony‐stimulating factor (G‐CSF) of 30 MU was administered, followed by an optimal response after 24 hours: white blood cells, 15.59 × 10^9^/L; absolute neutrophil count, 10.92 × 10^9^/L (Figure 1).

### 2.2 Follow‐up

Three days later, on April 17, she was discharged from the hospital while maintaining a normal ANC (2.10 × 10^9^/L) on day 41 of the infection (May 12).

## 3 DISCUSSION

Neutropenia is a common hematological finding. An appropriate clinical approach initiates with an exhaustive medical history (acute or chronic neutropenia, family history, drugs, and concomitant illnesses) and a physical examination. Regarding laboratory data, other cytopenias must be excluded, as well as autoimmune disorders based on a general antibody test, including several infectious diseases based on serological markers. In addition, vitamin B12, folate, and other vitamin levels must be measured. Peripheral blood smears must also be examined in all cases. In our patient, all these parameters were studied, showing no abnormalities. Therefore, given the severity of the neutropenia, a bone marrow examination was performed to exclude myelodysplasia, acute leukemias, marrow failure, and the occurrence of hemophagocytosis. Collectively, the most frequent causes of neutropenia in our case were ruled out, indicating SARS‐CoV‐2 as the most likely etiological agent. A potential explanation is that transient agranulocytosis occurred earlier with the infection, and the marrow was beginning to recover when the bone marrow aspirate was performed. Another explanation could be peripheral neutrophil consumption. An immune mechanism remains possible; however, given the rapid and sustained response to a single dose of G‐CSF, this seems unlikely.

The use of G‐CSF to prevent infections in cases of acute and chronic neutropenia is well established. Responses to G‐CSF are observed in autoimmune and idiopathic neutropenia.^3^ Our approach for using G‐CSF in this case with an ANC < 0.5 × 10^9^/L was implemented to test the bone marrow response to myeloid growth factor, shorten the period of severe neutropenia, and prevent bacterial co‐infections with COVID‐19.

Other viral infections are associated with the development of transient neutropenia, especially infections of human herpesvirus 6, parvovirus, EBV, adenovirus, influenza A, HIV, hepatitis C virus, and cytomegalovirus. Central and peripheral mechanisms may be involved. These viruses can cause direct damage to bone marrow progenitors and trigger autoimmune destruction. Neutropenia may sometimes occur secondary to antiviral treatment (such as zidovudine, ribavirin, and ganciclovir).^4^, ^5^, ^6^

Human coronaviruses frequently cause mild and self‐limited upper respiratory tract infections in adults.^7^ More severe clinical manifestations are frequently observed in immunocompromised patients.^8^ Although leukopenia with lymphopenia is a common finding, monocyte and neutrophil counts are within normal limits in most cases of human coronavirus infections.^9^

SARS‐CoV‐2 infection can cause several hematological abnormalities. Other cases of autoimmune cytopenias such as thrombocytopenia and hemolytic anemia have been described.^10^, ^11^ Lymphopenia is the most common laboratory finding in patients with COVID‐19. Low nadirs of lymphocyte and monocyte counts, as well as high peaks of absolute neutrophil counts, have been reported in severe cases of COVID‐19 that require admission of patients to the intensive care unit.^1^, ^2^, ^10^, ^11^ Neutrophilia predicts poor outcomes in patients with COVID‐19 and severe respiratory failure. Some authors have postulated the potential role of neutrophil extracellular traps (NETs) as an aberrant immune response in patients with increased neutrophil counts and SARS‐CoV‐2 infection. Excessive NET generation leads to a cascade of inflammatory reactions that destroy surrounding tissues and cause microthrombosis, damaging vital organs, especially the lungs, as it occurs in severe cases of COVID‐19.^12^, ^13^ Our case could illustrate a different subset of patients with mild disease, no respiratory symptoms, and neutropenia instead of neutrophilia.

To the best of our knowledge, this is the first case of acquired severe neutropenia in the context of COVID‐19 reported to date. Further studies with a greater number of patients are needed to explore the true incidence and mechanisms of neutropenia in patients with COVID‐19.

## CONFLICT OF INTEREST

The authors declare no conflicts of interest.

## AUTHOR CONTRIBUTIONS

All authors contributed equally to this manuscript.

## ETHICAL APPROVAL

The patient consent has been signed and collected in accordance with the journal's patient consent policy.
